# Vibrotactile stimulation for upper-limb neurorehabilitation and sensorimotor training: a systematic review of clinical and mechanistic evidence

**DOI:** 10.3389/fbioe.2026.1892452

**Published:** 2026-09-09

**Authors:** Wenbin Zhang, Kai Leng Catherine Chan, Hong Zeng, Baoguo Xu, Hexuan Hu, Minmin Miao, Aiguo Song, Ying Shen

**Affiliations:** 1 The College of Computer Science and Software Engineering, Hohai University, Nanjing, China; 2 Department of Rehabilitation Medicine, The First Affiliated Hospital with Nanjing Medical University, Nanjing, China; 3 School of Rehabilitation Medicine, Nanjing Medical University, Nanjing, China; 4 The State Key Laboratory of Digital Medical Engineering, School of Instrument Science and Engineering, Southeast University, Nanjing, China; 5 The School of Information Engineering, Huzhou University, Huzhou, China

**Keywords:** brain-computer interface, neurorehabilitation, sensorimotor, stroke, vibrotactile

## Abstract

**Background:**

Vibrotactile stimulation (VTS) has emerged as a potential sensory-based adjunct to upper limb rehabilitation by providing additional afferent feedback and supporting sensorimotor rehabilitation. This systematic review aimed to synthesize clinical evidence on VTS for post-stroke upper-limb rehabilitation and complementary mechanistic and technological evidence from healthy participants, with emphasis on intervention characteristics, stimulation parameters, clinical outcomes, neurophysiological effects, usability and safety.

**Methods:**

This review was conducted according to the Preferred Reporting Items for Systematic Reviews and Meta-Analyses (PRISMA) 2020 guidelines and was retrospectively registered with the Open Science Framework. PubMed, PEDro, Web of Science and Google Scholar were searched for English-language full-text studies published between January 2015 and January 2026. Search terms included combinations of stroke, VTS, vibration, tactile, somatosensory, haptic, upper limb and hand.

**Results:**

A total of 35 studies were included. Fourteen studies used direct upper-limb stimulation, six integrated VTS with robotic systems, two with virtual reality (VR), two with mirror visual feedback (MVF), seven with brain–computer interface (BCI) paradigms, and four with multimodal systems. Stimulation sites included the wrist, hand, fingers, fingertips, forearm, and upper arm, whereas reported frequencies, intensities, timing, and doses varied widely. Clinical studies suggested potential improvements in upper-limb motor function, sensory and proprioceptive function, spasticity, and affected-limb use, particularly when VTS accompanied active or task-oriented training. Mechanistic studies indicated that VTS may modulate sensorimotor rhythms, cortical activation, functional connectivity, and motor-imagery (MI)-related brain responses.

**Conclusion:**

Current evidence suggests that VTS may support upper-limb functional recovery after stroke. However, confidence in the evidence remains limited by small samples, heterogeneous protocols, and the frequent use of multimodal interventions that do not isolate the contribution of vibration. Further adequately powered randomized controlled trials are needed to determine clinical efficacy and to identify the stimulation parameters and feedback architectures most likely to benefit people after stroke.

## 1 Introduction

Stroke is the second leading cause of death and the third leading cause of death and disability combined worldwide, resulting in a substantial burden on individuals, families and society (Feigin et al., 2025). Upper limb dysfunction is a common and clinically significant consequence of stroke, often characterized by muscle weakness, spasticity and impaired motor coordination (Raghavan, 2015). These impairments can substantially restrict activities of daily living (ADL) that require upper limb control, including self-care, feeding and writing. Despite advances in neurorehabilitation, recovery of upper limb function remains challenging for many stroke survivors (Langhorne et al., 2011; Gittler and Davis, 2018). Therefore, improving upper limb recovery remains a major priority in neurorehabilitation research and clinical practice, particularly in post-stroke rehabilitation (Pollock et al., 2014; Veerbeek et al., 2014).

Post-stroke upper limb functional recovery is a complex process that depends not only on the restoration of muscle strength but also on effective sensorimotor integration (Krakauer, 2006; Borich et al., 2015). Sensorimotor integration enables the central nervous system (CNS) to combine sensory feedback from the affected limb with motor commands to plan, execute and adjust movement (Edwards et al., 2019). During upper limb rehabilitation, this process supports movement planning, motor relearning and cortical reorganization. However, many stroke survivors experience somatosensory deficits, including impaired tactile sensation and proprioception (Connell et al., 2008; Doyle et al., 2010; Meyer et al., 2014). These deficits may reduce the quality of sensory feedback from the paretic limb, thereby disrupting sensorimotor integration and limiting functional recovery. Therefore, peripheral sensory stimulation has gained increasing attention as a potential strategy to enhance sensory feedback and support sensorimotor integration during upper limb rehabilitation (Bolognini et al., 2016; Grant et al., 2018; Serrada et al., 2019; Asan et al., 2022). Various sensory stimulation modalities have been investigated to improve upper limb recovery after stroke, including electrical stimulation, thermal stimulation, proprioceptive training and visual feedback. Previous evidence suggests that sensory-based interventions can modulate cortical excitability, facilitate neuroplastic changes within sensorimotor networks and improve motor performance when combined with active rehabilitation training (Bolognini et al., 2016). Among these sensory-based approaches, vibration-based interventions offer several advantages, including non-invasive and precisely controlled sensory input, the ability to augment somatosensory feedback and facilitate sensorimotor integration, and compatibility with wearable devices and technology-assisted rehabilitation platforms.

**FIGURE 1:**
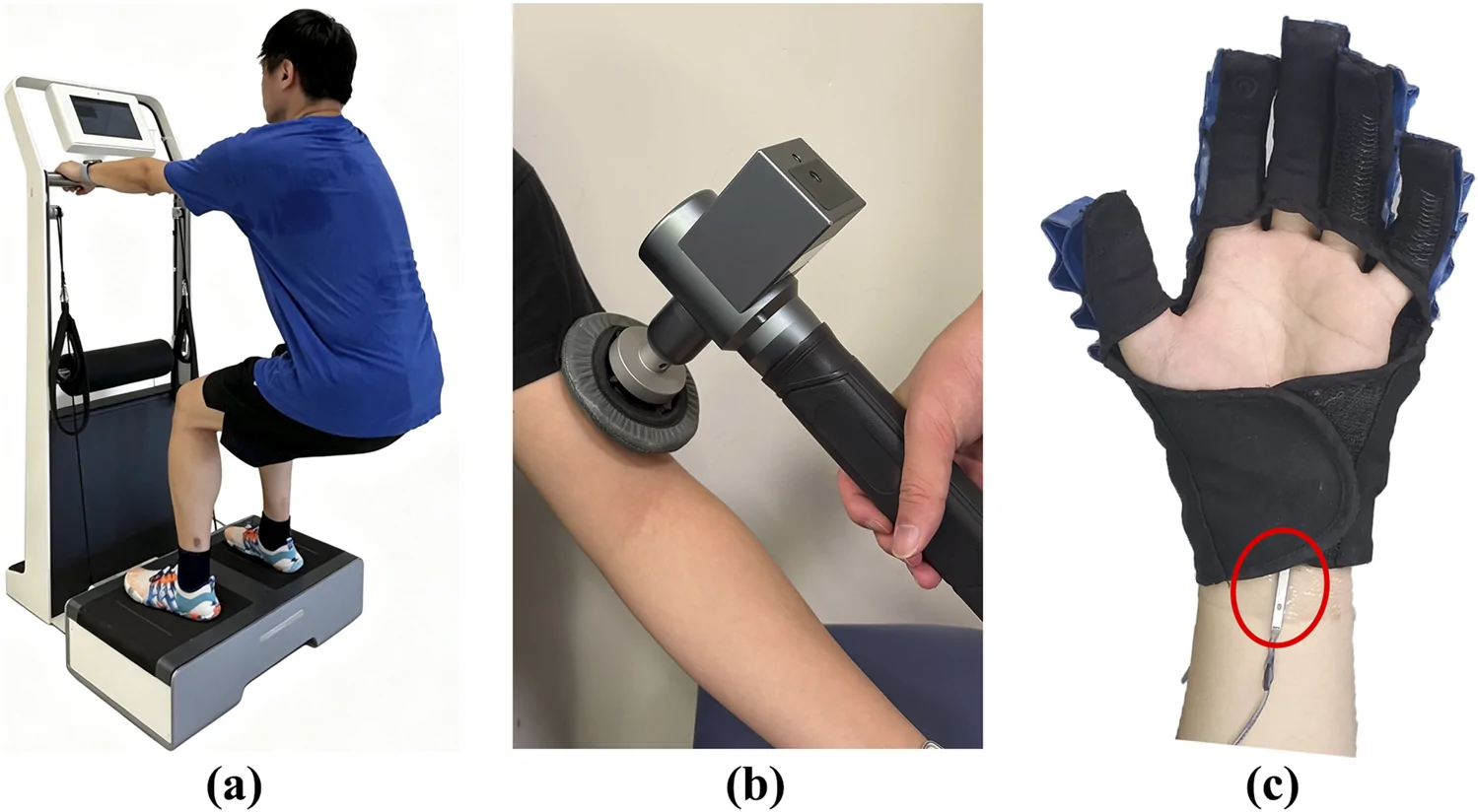
Comparison of vibration modalities: **(A)** whole-body vibration (WBV), **(B)** focal/localized muscle vibration (FMV/LMV), and **(C)** vibrotactile stimulation (VTS).

Accordingly, several forms of vibration-based interventions have been explored in neurorehabilitation to enhance sensory input and facilitate sensorimotor recovery (Lu et al., 2024). These approaches include whole-body vibration (WBV), localized muscle vibration (LMV) or focal muscle vibration (FMV) and vibrotactile stimulation (VTS). Although these approaches all use mechanical vibration, they differ in stimulation targets and delivery methods. WBV is typically delivered through a vibrating platform to induce systemic neuromuscular activation and has mainly been used for balance, gait and postural control (Lee, 2019; Sade et al., 2020). LMV or FMV is commonly applied directly to specific muscles or tendons to enhance muscle activation, alleviate spasticity, and induce movement illusions through proprioceptive stimulation, which may further facilitate sensorimotor processing (Giorgi et al., 2024; Le Franc et al., 2021). In contrast, VTS is typically delivered through localized or wearable actuators applied to the skin or selected upper limb regions. VTS may activate cutaneous mechanoreceptors and proprioceptive afferents without necessarily inducing overt movement, thereby providing tactile stimulation or task-related feedback during rehabilitation training (Johansson and Flanagan, 2009; Proske and Gandevia, 2012; Delhaye et al., 2018; Seim et al., 2021). This capacity for localized and task-related feedback supports the integration of VTS with robotic systems, virtual reality (VR), mirror visual feedback (MVF) and brain-computer interface (BCI). Within these systems, vibrotactile feedback can be synchronized with movement practice, motor imagery (MI) or virtual task performance to support upper limb rehabilitation after stroke. In robotic rehabilitation, VTS may serve as an additional sensory feedback modality that supports sensorimotor integration and motor learning through augmented afferent input (Basteris et al., 2014; Cuppone et al., 2016; Veerbeek et al., 2017). In VR, it can provide haptic feedback during interaction with virtual objects, thereby enhancing task engagement and sensorimotor feedback (Lin et al., 2016; Bae and Park, 2023; Laver et al., 2025; Bonnet et al., 2026). In MVF paradigms, VTS may enhance embodiment perception and increase motor cortical activation by combining visual illusion with tactile input (Thieme et al., 2013; Ding et al., 2020). In BCI-based rehabilitation, vibrotactile feedback during MI training may improve BCI-related neural responses by enhancing mu-rhythm desynchronization over the contralateral motor cortex and increasing motor cortical excitability (Shu et al., 2017, Shu et al. 2018; Cervera et al., 2018; Khan et al., 2020; Grigorev et al., 2021). Typical application methods of WBV, FMV/LMV, and VTS are shown in Figure 1.

**FIGURE 2:**
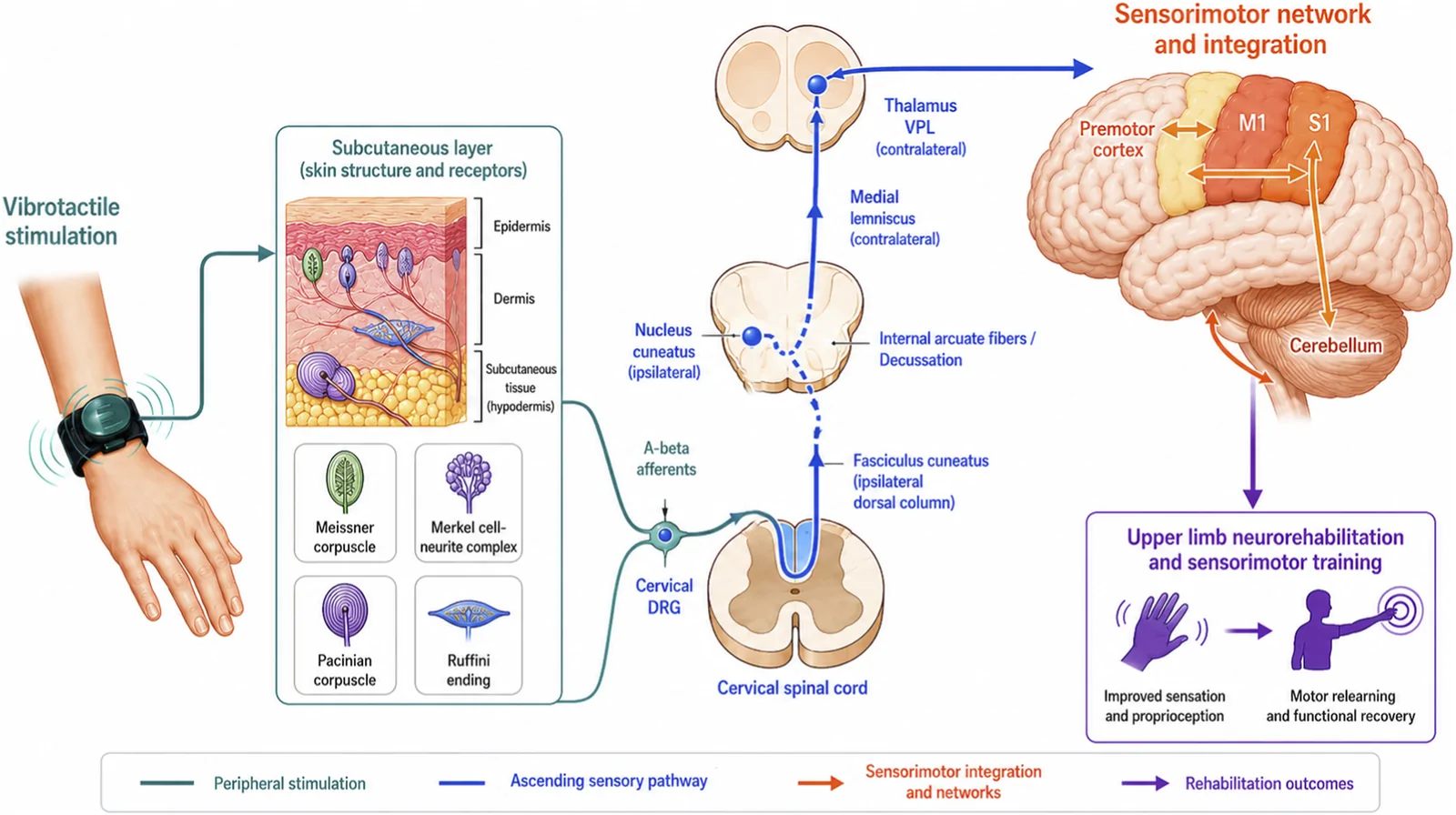
Peripheral and central neural pathways involved in vibrotactile perception.

The potential therapeutic effects of VTS may be associated with the modulation of peripheral sensory input and central sensorimotor processing (Seim et al., 2021). At the peripheral level, mechanical vibration may activate sensory receptors in the skin, muscles and other upper limb tissues, thereby increasing peripheral sensory input from the affected limb (Delhaye et al., 2018). Figure 2 shows the principal peripheral and central pathways involved in vibrotactile perception, from cutaneous mechanoreceptor activation and ascending somatosensory transmission to cortical sensorimotor processing. This increased sensory input may improve the quality of sensory feedback available for movement control and motor relearning. At the central level, enhanced sensory feedback may modulate central sensorimotor processing, including cortical excitability (de Freitas Zanona et al., 2023), sensorimotor network activation (Shu et al., 2018; Zhang et al., 2022; Herrera Altamira et al., 2023) and functional reorganization (Iwama et al., 2025). Through these peripheral and central mechanisms, VTS may strengthen sensorimotor integration, support motor relearning and contribute to upper limb functional recovery after stroke. Taken together, these mechanisms indicate that VTS may have therapeutic potential for improving upper limb function after stroke.

Although previous studies have reported the potential benefits of vibration-based interventions in neurorehabilitation, several gaps remain. Previous systematic and scoping reviews have evaluated vibration-based, sensory-priming, or broader somatosensory interventions for upper-limb rehabilitation after stroke (Yilmazer et al., 2019; Stoykov et al., 2022; Lu et al., 2024). However, these reviews have generally considered VTS as only one component within heterogeneous sensory interventions or have not distinguished it from other forms of mechanical vibration. Consequently, they provide limited information regarding the unique therapeutic role of VTS, its stimulation characteristics, integration with wearable technologies, and application within emerging rehabilitation platforms such as robotic systems, VR, MVF, and BCI systems.

Importantly, VTS differs from other vibration modalities in both its physiological targets and clinical applications. Rather than primarily inducing whole-body neuromuscular responses or focal muscle spindle activation, VTS delivers localized cutaneous sensory feedback that can be synchronized with motor tasks and incorporated into interactive rehabilitation technologies. Furthermore, the number of studies investigating VTS has increased substantially in recent years, particularly in technology-assisted rehabilitation. This rapid growth highlights the need for an updated systematic review that specifically evaluates VTS as a distinct therapeutic modality. Therefore, this systematic review focuses specifically on VTS as a distinct sensory intervention and aims to synthesize current evidence regarding its intervention characteristics, stimulation parameters, clinical and functional outcomes, neurophysiological effects, usability and safety across different upper limb rehabilitation paradigms.

## 2 Methods

### 2.1 Protocol and registration

This systematic review was not prospectively registered before the initial literature search. To improve methodological transparency, a review protocol specifying the eligibility criteria, outcomes of interest, data-extraction items, methodological quality assessment, and synthesis plan was subsequently registered with the Open Science Framework on 22 July 2026 (OSF; Registration DOI: 10.17605/OSF.IO/KYMJR).

### 2.2 Search strategy

This systematic review was conducted in accordance with the Preferred Reporting Items for Systematic Reviews and Meta-Analyses (PRISMA) 2020 guidelines (Page et al., 2021). PubMed, Web of Science Core Collection, the Physiotherapy Evidence Database (PEDro), and Google Scholar were systematically searched for studies published from 1 January 2015, to 30 January 2026. The search focused on vibrotactile stimulation or vibrotactile feedback applied to the upper limb in the context of post-stroke rehabilitation and related sensorimotor training.

The search terms covered four principal concepts: vibrotactile stimulation and feedback, upper-limb applications, stroke rehabilitation, and sensorimotor or neurophysiological processes. Terms related to vibrotactile stimulation, such as “vibrotactile stimulation,” “vibrotactile feedback,” “tactile vibration,” “cutaneous vibration,” “vibratory stimulation,” and “haptic feedback,” were combined with upper-limb terms, including “upper limb,” “upper extremity,” “arm,” “forearm,” “wrist,” “hand,” and “finger.” These terms were further combined with stroke- and rehabilitation-related terms, including “stroke,” “post-stroke,” “hemiparesis,” “hemiplegia,” “rehabilitation,” and “neurorehabilitation,” or with terms related to sensorimotor training and underlying mechanisms, including “sensorimotor,” “somatosensory,” “proprioception,” “motor imagery,” “brain-computer interface,” “robot-assisted rehabilitation,” “virtual reality,” “mirror visual feedback,” “electroencephalography,” and “cortical activity.”

Two complementary search combinations were used. The first combined vibrotactile, upper-limb, stroke, and rehabilitation-related terms to identify studies involving individuals with stroke. The second combined vibrotactile and upper-limb terms with sensorimotor, neurophysiological, and technology-assisted training terms. Stroke-related terms were not mandatory in the second combination because some studies relevant to the sensory, neural, and technological basis of upper-limb rehabilitation were conducted in healthy participants. Boolean operators, phrase searching, truncation, controlled vocabulary, and database-specific field restrictions were applied where supported.

Because PEDro and Google Scholar do not support the same complex search syntax as PubMed and Web of Science, several shorter combinations of the principal terms were used on these platforms. The Google Scholar search was limited to the predefined publication period, and retrievable results were screened in relevance order.

No full-text availability filter was applied during database searching. Only full-text articles published in English were included during eligibility assessment. The reference lists of the included articles and relevant reviews were manually examined, and forward citation searching was conducted to identify additional studies.

### 2.3 Study selection

All retrieved records were imported into Zotero version 9.0.6 (Corporation for Digital Scholarship, 2026). Duplicate records were identified using Zotero’s automated duplicate-detection function and subsequently verified manually. Studies were eligible if they met the following criteria: (1) the participants were individuals with stroke or healthy adults involved in an upper-limb sensorimotor experiment relevant to stroke rehabilitation; (2) vibrotactile stimulation or vibrotactile feedback was applied to the upper limb or delivered in relation to an upper-limb task; (3) the study reported at least one motor, sensory, proprioceptive, neurophysiological, behavioral, rehabilitation-related, usability, or safety outcome; (4) original empirical data were reported; and (5) the full-text article was published in English.

For this review, VTS was defined as externally generated mechanical vibration delivered through direct contact with the skin or an upper-limb interface and intended to provide tactile, cutaneous, or task-related sensory input. Muscle- or tendon-adjacent vibration was retained only when it formed an integral, task-synchronized component of an MI paradigm or a multimodal upper-limb rehabilitation system. Studies whose primary purpose was isolated focal muscle or tendon vibration to elicit a tonic vibration reflex, facilitate contraction, or induce a kinaesthetic illusion were excluded. Studies using only non-vibratory force, pressure, or resistance feedback were also excluded. Healthy-participant studies were eligible only when the protocol, outcome, or technology-assisted task was directly relevant to the sensory, neural, or technical basis of upper-limb rehabilitation after stroke.

Studies were excluded if they: (1) involved patient populations other than stroke without separately reported stroke data; (2) applied stimulation exclusively to the lower limb, trunk, or whole body; (3) investigated isolated focal muscle or tendon vibration primarily to induce a tonic vibration reflex, muscle contraction, or kinaesthetic illusion; (4) used only non-vibratory force, pressure, or resistance feedback; (5) did not include an identifiable vibrotactile or vibration-feedback component; (6) focused on consumer haptics, entertainment, communication, or general human-computer interaction without clear relevance to upper-limb rehabilitation or sensorimotor training; (7) were reviews, editorials, protocols, conference abstracts without sufficient original data, or other non-empirical publications; or (8) were not available as full-text articles in English.

After duplicate removal, two reviewers independently screened the titles and abstracts of all 118 unique records against the predefined eligibility criteria. Records considered potentially eligible by either reviewer were retained for full-text assessment. The same two reviewers independently assessed 60 full-text reports and recorded reasons for exclusion. At each stage, the reviewers recorded an initial binary decision (retain or exclude at title-and-abstract screening; include or exclude at full-text assessment) before discussion. Percentage agreement and Cohen’s kappa were calculated separately for the two screening stages.

**FIGURE 3:**
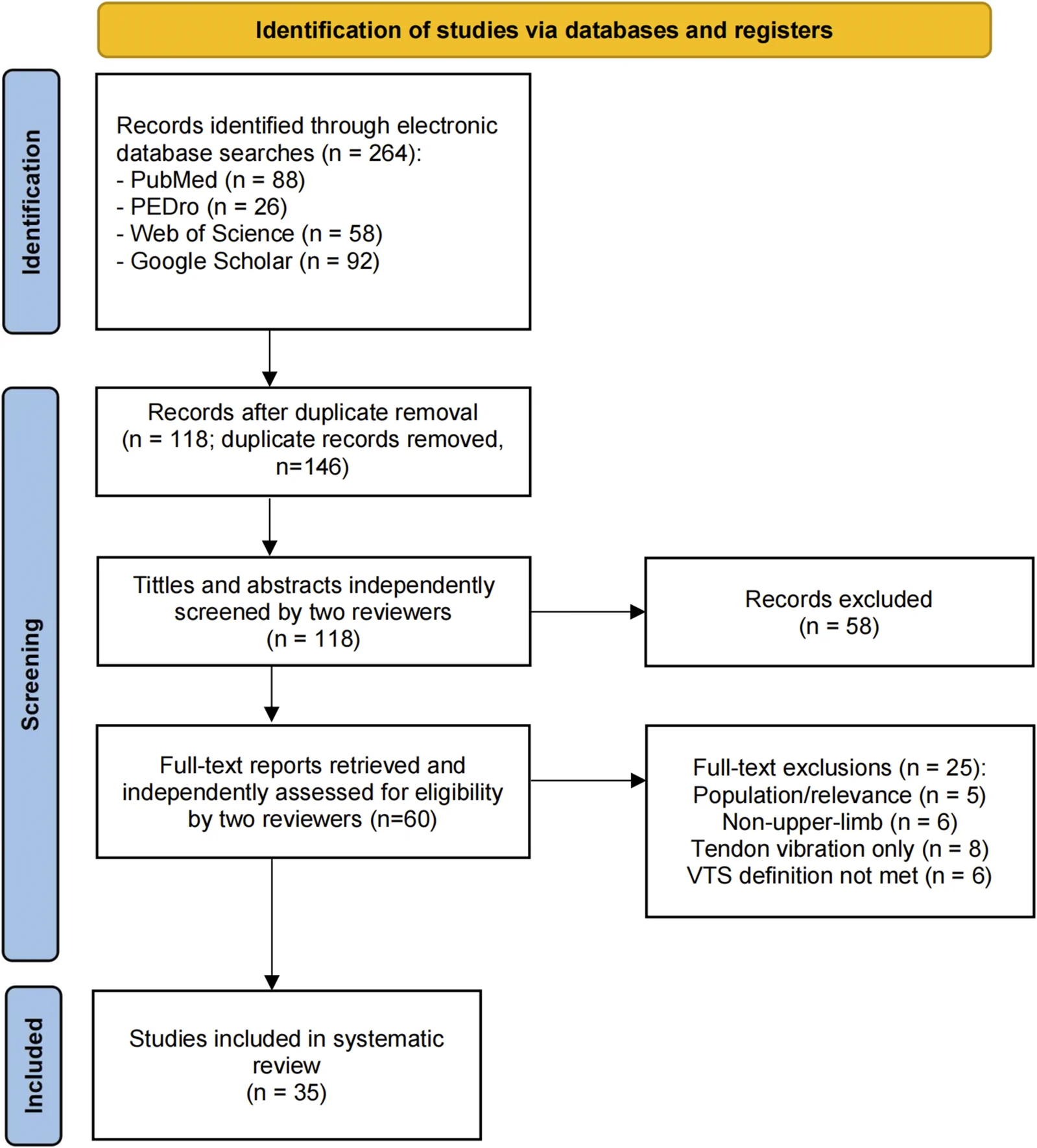
PRISMA 2020 flow diagram of study selection.

No disagreement occurred during title-and-abstract screening. Disagreements at full-text assessment were resolved through discussion, and no third-reviewer adjudication was required. The study selection process was summarized using a PRISMA flow diagram (Figure 3) and the eligibility criteria based on the population, intervention, comparison, and outcome (PICO) framework are presented in Table 1.

**TABLE 1:** PICO framework.

Characteristics	Criteria
Population	Human participants, including healthy individuals and individuals with stroke
Intervention	Upper limb vibrotactile stimulation or vibrotactile feedback
Comparison	Conventional rehabilitation, sham stimulation, no stimulation, alternative feedback, or baseline/pre-intervention condition
Outcomes	Upper limb motor function, sensory function, spasticity, activities of daily living, pain, neurophysiological responses, usability, and adverse events

### 2.4 Data extraction

Data were extracted from each included study and are summarized in Table 2. Extracted items included author and year, participant population, study design, application mode, feedback architecture, stimulation frequency or frequency range, intensity when reported, stimulation site, device or actuator, outcome measures, and main findings. Application modes were classified as direct stimulation, robot-assisted VTS, VR-based VTS, MVF-related VTS, BCI-related VTS, or multimodal systems. Feedback architectures were classified as open-loop or background stimulation, task-synchronized cueing, real-time state/error/performance feedback, or adaptive neural closed-loop feedback. Missing information was recorded as “NR”. Disagreements were resolved through discussion and review of the original full text.

**TABLE 2:** Study designs, participants, vibrotactile systems, feedback architectures, and outcomes of the included studies. Item-level methodological-quality judgments are reported separately in Table 3.

Study	Participants and design	Application	Feedback mode	Frequency/intensity	Site	Device/actuator	Outcomes	Main findings
Direct VTS								
Alouit et al. (2025)	44 healthy adults (22 per experiment); two independent EEG experiments	Direct stimulation	Open-loop, frequency-coded stimulation	10, 50, and 250 Hz	Fingers	Finger stimulation setup	SEPs; cortical connectivity; source localization	VTS produced delayed SEPs but stronger early local connectivity than electrical stimulation
Kitai et al. (2021)	1 stroke survivor; 6-week single-case intervention	Direct stimulation	Real-time contact-vibration feedback	Not measured	Fingers	Yubi-recorder (tech giken Co., Ltd.).	Position sense; NRS; FMA; STEF; MAL; EEG.	Single-case results showed improved FMA, STEF, numbness, MAL and agency scores
Lakshminarayanan et al. (2017)	10 chronic stroke survivors; usability study	Direct stimulation	Continuous subsensory open-loop stimulation	Not measured; random-frequency white-noise command with 500-Hz low-pass cutoff; 60% sensory threshold	Wrist	TheraBracelet prototype	Expectation/satisfaction surveys; prototype experience	Participants accepted the low-cost orthotic concept; satisfaction averaged 3.7/5
Mazorow et al. (2024)	15 healthy young adults; 3-day within-subject training	Direct stimulation	Real-time 3D limb-state feedback	Not measured; actuator operating range 90–200 Hz	Arm	Experimental VTF system using six ERM motors (precision microdrives 310–117)	Target capture error/speed; SUS; motivation; QUEST.	Training reduced target error, but concurrent VTF slowed movement; usability and motivation were positive
Risi et al. (2019)	15 healthy adults; 2-day within-subject reaching study	Direct stimulation	Real-time 2D limb-position feedback	Not measured; actuator operating range 50–250 Hz (approximately 60–240 Hz used)	Arm	Two-dimensional vibrotactile display/interface	Reach accuracy/precision; proprioceptive drift; hand path	VTF improved reach accuracy over 2 days but increased cognitive/strategy demands
Schranz et al. (2022)	12 chronic stroke survivors; secondary EEG analysis of a pilot RCT [a]	Direct stimulation	Subsensory background stimulation during task practice	Not measured; random-frequency command; 60% sensory threshold	Wrist	NR	EEG connectivity/coherence; ERD/ERS; BBT.	Treatment increased EEG connectivity versus control and shifted ERD/ERS patterns
Scronce et al. (2023)	12 chronic stroke survivors; double-blind pilot RCT	Direct stimulation	Subsensory background stimulation plus home exercise	Not measured; random-frequency command; 60% sensory threshold	Wrist	TheraBracelet.	Home-exercise adherence; wear logs; SIS-Hand; FMA; BBT.	Home-exercise adherence varied widely; greater cumulative practice was associated with larger SIS-Hand gains in the stimulation group
Seim et al. (2023)	14 stroke survivors; sham-controlled randomized crossover	Direct stimulation	Acute open-loop site comparison	67–70 and 253–255 Hz	Fingertips	Custom vibration motors (precision microdrives models #310–103 and #C08-005)	MAS; MTS; finger pressure	Cutaneous fingertip stimulation significantly reduced MAS and MTS; sham did not
Seim et al. (2021)	16 chronic stroke survivors; double-blind pilot RCT	Direct stimulation	Patterned open-loop home stimulation	Approx. 175 Hz on-glove (approx. 210 Hz unloaded); approx. 1.3 g	Fingertips	VTS glove with ERM motors (precision microdrives 310–113)	SWMT; MAS; AROM; adherence/usability	VTS improved tactile perception, reduced finger MAS, and increased selected upper-limb active ranges of motion
Seo et al. (2019)	12 chronic stroke survivors; triple-blind pilot RCT [a]	Direct stimulation	Subsensory background stimulation during therapy	Not measured; random-frequency white-noise command with 500-Hz low-pass cutoff; 60% sensory threshold	Wrist	TheraBracelet; C-3 tactor vibrator	BBT; WMFT hand-task time; sensory threshold; tolerability	BBT improved after therapy and remained higher at follow-up
Seo et al. (2020)	26 enrolled/25 completed chronic stroke survivors; double-blind crossover safety trial	Direct stimulation	Continuous subsensory home stimulation	NR; random-frequency vibration; 60% threshold real, 1% sham	Wrist	TheraBracelet/wristwatch device with C-3 tactor vibrator	AEs; adherence; NHPT; grip strength; pain; MAS; skin status	Daily stimulation was safe and well tolerated; wear time averaged about 10 h/day
Signal et al. (2020)	20 stroke survivors; multiple-period randomized crossover [b]	Direct stimulation	Scheduled open-loop haptic nudge	150 Hz	Wrist	BuzzNudge wearable device	Observed affected and unaffected upper-limb movements	Planned haptic nudges increased affected upper-limb movement odds
Signal et al. (2023)	Same 20-participant cohort; secondary temporal analysis [b]	Direct stimulation	Scheduled nudge; timing-response analysis	150 Hz	Wrist	BuzzNudge wearable device	Observed affected upper-limb movement by 10-s intervals	Affected arm use increased immediately after nudges, strongest during afternoon activity
Wu et al. (2017)	9 chronic stroke survivors; uncontrolled pre-post feasibility study	Direct stimulation	Task-synchronized game cue	Not measured	Fingers	Vibrotactile glove rehabilitation system	Finger ROM; strength; NHPT; MHQ; satisfaction	Hand measures showed modest nonsignificant gains; satisfaction was positive
Robot-assisted VTS								
Amano et al. (2020)	6 chronic stroke survivors; uncontrolled multimodal feasibility trial	Robot + VTS	Task-coupled vibration in a multimodal robot	100 Hz	Upper limb	Yaskawa reaching robot with NMES and FM34F motor	FMA-UE; ARAT; MAS; reaching kinematics	Multimodal training was feasible; FMA-UE, ARAT, and elbow active range improved, but the VTS contribution was not isolated
Cuppone et al. (2016)	28 healthy adults; 3-day controlled training study	Robot + VTS	Real-time movement-error feedback	70, 80, or 90 Hz	Forearm	WristBot plus wearable VTF setup	Wrist position matching; tracking; reliance on guidance	Position-sense acuity improved and reliance on guidance decreased; tracking transfer was limited
Du et al. (2022)	14 healthy adults; within-subject fNIRS experiment	Robot + VTS	Task-synchronized multimodal stimulation	100 Hz; approximately 0.5 N	Fingertips	Vibrotactile enhanced pneumatically actuated hand rehabilitation device	fNIRS HbO; lateralization; effective connectivity	Vibrotactile enhancement increased sensorimotor activation/connectivity in healthy participants
Rodríguez-Pérez et al. (2022)	18 stroke survivors; single-blind non-randomized controlled trial	Robot + VTS	Robot-integrated open-loop vibration	60 Hz; intensity adjusted to tolerance	Fingertips	Amadeo system version 5.1	SWM; FMA-UE; MAL-14; SIS-16	Robot-assisted vibration plus conventional therapy improved sensory and motor measures within group
Yeh et al. (2021)	12 chronic stroke survivors +10 matched healthy controls; pre-post/retention study	Robot + VTS	Real-time ball position/speed feedback	80, 90, and 100 Hz	Forearm	WristBot robotic wrist/hand exoskeleton with VTF motors	Wrist position-sense JND; tracing error; SEPs	JND thresholds improved after training and at retention; transfer to tracing was modest
Yunus et al. (2020)	14 participants with upper-limb disability; psychophysical system test	Robot + VTS	Real-time finger/force encoding	Not measured; estimated motor range approximately 95–240 Hz and 0.2–0.65 g	Upper limb	Vi-HaB wearable vibrotactile haptic feedback system	Psychophysical accuracy for finger awareness and force feedback	Accuracy reached 82.0%, exceeding the preset performance threshold
VR-based VTS								
Bae and Park (2023)	11 healthy adults +1 stroke survivor; single-session fNIRS pilot	VR + VTS	Performance-contingent VR feedback	Not measured; device output range 50–160 Hz	Wrist	Meta quest 2; C2-HDLF vibrotactors; Unity/Oculus; fNIRS validation	Gesture success; fNIRS activation; user experience	The VR-VTF task increased HbO relative to rest; the VTS-specific contribution was not isolated
Lin et al. (2016)	4 healthy adults; technical feasibility test	VR + VTS	Task-synchronized finger cue	200 Hz	Hand or fingers	Novel haptic rehabilitation glove/vibration-assisted glove	Pressure threshold; hit/error rate; reaction time	No errors occurred in the tested window; reaction time decreased with practice
MVF-related VTS								
Ding et al. (2020)	12 healthy adults; within-subject EEG pilot	MVF + VTS	Task-synchronized MVF cue	27–175 Hz	Hand	C10-100 LRA in elastic band; mirror holder; wireless EEG.	Embodiment latency/questionnaire; EEG ERD/ERS; network metrics	Congruent or incongruent VTS strengthened embodiment and alpha-band motor cortical activation
Oliveira et al. (2018)	21 stroke survivors (7/group); three-arm randomized pilot	MVF + VTS	Open-loop continuous stimulation	35 Hz; 1.5-mm amplitude	Upper limb	Digital Vibration Pad (nissan fisio, sao paulo, Brazil)	RMI; WMFT; JTT.	Mirror therapy or vibration therapy improved upper-limb function after stroke
BCI-related VTS								
Adham et al. (2024)	16 healthy adults; within-subject EEG experiment	BCI + VTS	Task-synchronized tendon-adjacent feedback	70–100 Hz	Right extensor radialis longus carpi tendon	Vibrasens device (VB 115, Techno Concept™)	EEG beta ERD	Vibration increased left sensorimotor recruitment and embodiment; visual and vibratory effects were partly additive
Chen et al. (2019)	11 younger +11 older healthy adults; age-group comparison	BCI + VTS	Open-loop vibrotactile stimulus	27–175 Hz	Wrist	(type C10–100, Precision Microdrivers Ltd.)	ERSP/ERD; BCI offline accuracy	Age affected stimulation-related cortical activation; older participants showed reduced lateralization
Grigorev et al. (2021)	10 healthy adults; within-subject MI-BCI/nTMS study	BCI + VTS	BCI-contingent vibrotactile neurofeedback	Not measured; 500-Hz pulse rate and 1.2-ms pulse width	Forearm and back of neck	Flat LRA, 3 V, 10 mm diameter; Arduino Nano microcontroller; nTMS and EEG systems used for assessment	BCI accuracy; ERD; nTMS MEPs	VTF increased contralateral mu ERD and motor-cortical excitability, but did not significantly change BCI accuracy
Shu et al. (2017)	22 healthy adults +4 stroke patients; enhanced vs. control groups	BCI + VTS	Task-synchronized unilateral cue	27–175 Hz	Wrist	C10-100 LRA; audio amplifier; custom wrist band	ERSP/ERD; online/offline BCI accuracy; R2	Unilateral tactile stimulation enhanced contralateral activation during MI of the stimulated hand
Shu et al. (2018)	11 stroke patients; within-subject online BCI study	BCI + VTS	Task-synchronized cue during motor attempt	Not measured	Wrist	Precision microdrives DC motor; Arduino; custom wrist band	Online/offline BCI accuracy; ERSP; R^2^	TS-MA improved online and offline BCI accuracy compared with MA alone
Yakovlev et al. (2023)	20 healthy adults enrolled/17 analyzed; EEG experiment	BCI + VTS	Open-loop stimulation/tactile imagery	Not measured; variable-frequency command	Arm	Arduino UNO	Time frequency analysis; ERD/ERS	Real tactile stimulation and tactile imagery induced contralateral mu ERD; beta ERD was evident mainly during real stimulation
Zhang et al. (2022)	18 healthy adults; three-condition within-subject MI-BCI study	BCI + VTS	EEG-phase-dependent adaptive closed loop	23–200 Hz	Wrist	Piezo actuators; audio amplifier; MATLAB phase prediction	Classification accuracy; ERSP/ERD; PLV.	Phase-dependent VTS enhanced sensorimotor activation and functional connectivity during MI.
Multimodal systems								
Batista et al. (2024)	19 healthy adults; HMD/screen x with/without VTF experiment	BCI + VR + VTS	Task-synchronized VR/BCI feedback	Not measured	Hand	NeuRow VR-BCI paradigm; Oculus Rift CV1 HMD; Oculus Rift hand controllers; LiveAmp EEG.	Alpha/beta ERD; lateralization index	VTF with embodied VR produced stronger alpha ERD; lateralization indices did not differ significantly
Lakshminarayanan et al. (2024)	12 healthy adults; BCI-virtual robot prototype study	BCI + VR + robot + VTS	BCI-controlled virtual robot with concurrent VTS	Not measured; 0–500-Hz white-noise command spectrum	Wrist	OpenBCI/OpenVibe; vibration motors; Unity virtual robot	BCI accuracy; confusion metrics; virtual robot control	Virtual robot control was feasible, with a mean true-positive rate of approximately 63.3%; clinical efficacy was not tested
Ramu and Lakshminarayanan (2023)	10 healthy adults; with/without pre-MI VTS experiment	BCI + robot + VTS	Brief pre-MI vibrotactile priming	Not measured; 0–500-Hz white-noise command spectrum; 150-ms pre-cue	Fingertips	Flat vibration micro motors, SunRobotics; custom sensory stimulation box with adjustable motor sliders	Mu/beta ERD; digit-classification accuracy	Brief pre-MI vibration increased mu/beta ERD and improved within-hand digit classification
Zhang et al. (2026)	23 healthy adults +5 stroke patients; EEG/fNIRS pilot (published 2026)	BCI + robot + VTS	BCI-driven robotic training with task-synchronized VTS	200 Hz	Wrist	Vibrotactile-assisted brain-controlled soft robotic hand system	Online MI decoding; EEG/fNIRS activation; functional connectivity	VTS enhanced sensorimotor activation, improved online decoding mainly in poor baseline performers, and strengthened bilateral sensorimotor activation in five stroke patients

Abbreviations: BCI, brain–computer interface; BBT, box and block test; EEG, electroencephalography; ERD/ERS, event-related desynchronization/synchronization; fNIRS, functional near-infrared spectroscopy; HMD, head-mounted display; MA, motor attempt; MI, motor imagery; MVF, mirror visual feedback; NR, not reported; RCT, randomized controlled trial; VTF, vibrotactile feedback; VTS, vibrotactile stimulation. [a] Schranz et al. (2022) is a secondary analysis of the Seo et al. (2019) trial. [b] Signal et al. (2020); Signal et al. (2023)) analyzed the same participant cohort.

### 2.5 Methodological quality appraisal

Methodological quality was appraised using the Mixed Methods Appraisal Tool (MMAT), version 2018 (Hong et al., 2018), which accommodates heterogeneous empirical study designs. Each report was assigned to the appropriate quantitative randomized, quantitative non-randomized, or quantitative descriptive category and assessed against the five corresponding design-specific criteria. Two reviewers independently appraised each report, and disagreements were resolved through discussion and re-examination of the full text. Judgments were recorded as “Yes”, “No”, or “Can’t tell” when reporting was insufficient. In accordance with MMAT guidance, no global numerical score was calculated; item-level judgments and the principal methodological concern for each report are presented in Table 3.

**TABLE 3:** MMAT 2018 item-level methodological-quality appraisal of the included reports.

Study	Category	C1	C2	C3	C4	C5	Principal methodological concern
Adham et al. (2024)	NRS	CT	Y	Y	Y	Y	Convenience healthy sample; indirect clinical evidence
Alouit et al. (2025)	NRS	CT	Y	Y	N	Y	Separate experimental samples limit direct modality comparison
Amano et al. (2020)	NRS	Y	Y	Y	N	Y	Uncontrolled, multimodal pre–post intervention
Bae and Park (2023)	NRS	CT	Y	Y	N	Y	Mixed pilot sample; VTS effect not isolated from the VR task
Batista et al. (2024)	NRS	CT	Y	Y	Y	Y	Convenience healthy sample; motor-execution condition was always last
Chen et al. (2019)	NRS	CT	Y	Y	N	Y	Age-group comparison with limited control of other group differences
Cuppone et al. (2016)	RCT	CT	Y	Y	CT	Y	Random-sequence generation, concealment, and assessor blinding insufficiently reported
Ding et al. (2020)	NRS	CT	Y	Y	Y	Y	Small healthy mechanistic sample
Du et al. (2022)	NRS	CT	Y	Y	CT	Y	Task-order and confounding control insufficiently reported
Grigorev et al. (2021)	NRS	CT	Y	Y	N	Y	Small healthy sample; session and training effects not fully controlled
Kitai et al. (2021)	QDS	Y	N	Y	Y	Y	Single-case report without a counterfactual
Lakshminarayanan et al. (2017)	QDS	Y	CT	CT	Y	Y	Small usability sample and incompletely validated survey measures
Lakshminarayanan et al. (2024)	QDS	Y	N	Y	Y	Y	Healthy prototype sample not representative of the intended clinical population
Lin et al. (2016)	QDS	CT	N	CT	Y	CT	Four-person technical test with limited sampling and analysis detail
Mazorow et al. (2024)	NRS	CT	Y	Y	N	Y	Uncontrolled training and practice effects
Oliveira et al. (2018)	RCT	CT	CT	Y	CT	CT	Randomization, baseline comparability, blinding, and adherence incompletely reported
Ramu and Lakshminarayanan (2023)	NRS	CT	Y	Y	Y	Y	Small healthy sample despite counterbalanced condition order
Risi et al. (2019)	NRS	CT	Y	Y	Y	Y	Convenience healthy sample; limited clinical generalizability
Rodríguez-Pérez et al. (2022)	NRS	Y	Y	Y	Y	Y	Non-randomized allocation and small sample despite adjusted analysis
Schranz et al. (2022)	RCT	CT	Y	Y	Y	Y	Secondary analysis of a very small pilot RCT.
Scronce et al. (2023)	RCT	CT	Y	N	Y	N	Three of 12 participants discontinued; home-exercise adherence was low and variable
Seim et al. (2021)	RCT	CT	N	Y	Y	Y	Important baseline functional imbalance in a small trial
Seim et al. (2023)	RCT	Y	Y	Y	N	Y	Outcome proctors could identify the administered stimulation condition
Seo et al. (2019)	RCT	CT	Y	Y	Y	Y	Very small pilot RCT; random-sequence generation and concealment incompletely reported
Seo et al. (2020)	RCT	Y	Y	Y	Y	Y	Primarily a safety trial; efficacy inference remains limited
Shu et al. (2017)	RCT	CT	CT	Y	CT	Y	Randomization and assessor blinding insufficiently reported; stroke subgroup extremely small
Shu et al. (2018)	NRS	Y	Y	Y	Y	Y	Small within-participant stroke study with surrogate BCI outcomes
Signal et al. (2020)	RCT	Y	Y	Y	N	N	Observer unblinded and scheduled nudges were sometimes not delivered
Signal et al. (2023)	RCT	Y	Y	Y	N	N	Secondary analysis with protocol deviations and the same cohort as Signal et al. (2020)
Wu et al. (2017)	NRS	Y	Y	Y	N	Y	Uncontrolled small pre–post feasibility study
Yakovlev et al. (2023)	NRS	CT	Y	Y	CT	Y	Three participants excluded; condition-order and confounding control incompletely reported
Yeh et al. (2021)	NRS	Y	Y	Y	N	Y	No no-VTF training control; practice and time effects remain possible
Yunus et al. (2020)	QDS	CT	N	CT	CT	CT	Small system-validation sample with limited measurement validation and reporting
Zhang et al. (2022)	NRS	CT	Y	Y	N	Y	Fixed condition sequence makes order effects plausible
Zhang et al. (2026)	NRS	CT	Y	Y	N	Y	Fixed session order, multimodal intervention, and five-person stroke subgroup

Abbreviations: CT, can’t tell because reporting was insufficient; MMAT, mixed methods appraisal tool; N, no; NRS, quantitative non-randomized study; QDS, quantitative descriptive study; RCT, quantitative randomized controlled trial; Y, yes. For RCTs, C1–C5 assess randomization, baseline comparability, complete outcome data, assessor blinding, and adherence. For NRSs, C1–C5 assess representativeness, measurement appropriateness, complete outcome data, control of confounding, and intervention fidelity. For QDSs, C1–C5 assess sampling relevance, representativeness, measurement appropriateness, nonresponse bias, and analysis appropriateness. No global score was calculated, in accordance with MMAT guidance.

## 3 Results

### 3.1 Study identification

A total of 264 records were identified through electronic database searches, including PubMed, PEDro, Web of Science, and Google Scholar. After duplicate removal, 118 records remained for title-and-abstract screening. The reviewers agreed on all 118 initial decisions (60 retain and 58 exclude), corresponding to 100% agreement and Cohen’s κ=1.000 . The full texts of the 60 retained reports were retrieved and independently assessed. At full-text assessment, the reviewers agreed on 55 of 60 initial decisions (34 include and 21 exclude), corresponding to 91.7% agreement. Five decisions were discordant: four reports were initially included only by reviewer 1 and one only by reviewer 2. Cohen’s κ for full-text assessment was 0.826. Following discussion, the four reports identified only by reviewer 1 were excluded and the report identified only by reviewer 2 was included. Thus, 25 full-text reports were excluded: five because the population or rehabilitation relevance was insufficient, six because they did not investigate the upper limb, eight because they examined isolated tendon vibration, and six because the intervention did not meet the review definition of VTS. Thirty-five studies were included in the final synthesis. The study-selection process is presented in Figure 3.

### 3.2 Study characteristics

The main characteristics of the included studies are summarized in Table 2. Thirty-five studies published between 2016 and 2026 were included. The studies showed substantial heterogeneity in design, participant population, application mode, feedback architecture, stimulation site, stimulation parameters, comparator, and outcome measures. Study designs included randomized or controlled clinical studies, non-randomized trials, crossover and within-subject experiments, pilot and feasibility studies, a single-case study, and neurophysiological investigations.

The evidence set comprised clinical studies involving stroke survivors and complementary mechanistic or feasibility studies involving healthy participants, including stroke-only, healthy-only, and mixed samples. Overall, 19 reports included at least one participant with stroke. Stroke-related studies primarily investigated motor function, sensory function, proprioception, spasticity, affected-limb use, safety, or rehabilitation feasibility, whereas healthy-participant studies mainly examined vibrotactile perception, sensorimotor mechanisms, neural responses, and technology integration.

Report-level sample sizes ranged from 1 to 44 participants, with a mean of 16.3 (SD 8.0), a median of 15, and an interquartile range of 12–20. The sum of reported enrolled samples was 569; however, this was not the number of independent participants because Signal et al. (2020), Signal et al. (2023) and Seo et al. (2019)/Schranz et al. (2022) reported overlapping cohorts. Most studies used small or pilot-scale samples, and long-term follow-up was uncommon. Because the studies differed substantially in population, intervention contrast, feedback architecture, comparator, and outcome domain, a structured narrative synthesis was performed instead of a meta-analysis.

### 3.3 Methodological quality

All 35 reports met the two MMAT screening questions. Eleven were appraised as quantitative randomized studies, 19 as quantitative non-randomized studies, and five as quantitative descriptive studies. Among the randomized reports, seven did not provide enough information to confirm appropriate sequence generation and allocation concealment; outcome-assessor blinding was absent in three and unclear in three. Among the non-randomized reports, sample representativeness was unclear in 14 of 19, and confounding was not adequately controlled in ten and was unclear in two. The descriptive feasibility reports were mainly limited by non-representative samples and incomplete reporting of sampling or measurement procedures. These concerns were considered when interpreting the consistency and clinical relevance of the findings; no report was excluded solely on the basis of methodological quality. Full judgments are provided in Table 3.

### 3.4 Application directions of VTS and stimulation parameters

The included studies were organized into six application groups: direct upper-limb stimulation (14 studies), robot-assisted VTS (six), VR-based VTS (two), MVF-related VTS (two), BCI-related VTS (seven), and multimodal systems combining more than one rehabilitation technology (four). In direct-stimulation studies, VTS was delivered to the hand, fingers, wrist, forearm, or upper arm without being tightly coupled to a robotic, virtual, mirror, or BCI platform. Wearable and direct systems served different functions, including background sensory stimulation, real-time limb-state feedback, acute spasticity modulation, and scheduled prompts intended to increase affected-limb use.

Across the 35 reports, feedback architectures were classified as open-loop or background stimulation (13 reports), task-synchronized cueing or stimulation (11), real-time state, error, or performance feedback (seven), and adaptive neural closed-loop feedback (four). Actuation technologies included eccentric rotating mass and linear resonant motors, C-3 tactors, piezoelectric actuators, and vibration modules integrated into wearable or robotic devices. Open-loop and task-synchronized systems predominated, whereas only four reports updated stimulation using neural signals or algorithmic states, indicating that evidence for adaptive closed-loop VTS remains limited.

Several studies combined VTS with robot-assisted rehabilitation. Cuppone et al. (2016) examined robot-assisted proprioceptive training with added vibrotactile feedback in healthy participants and reported enhanced somatosensory and motor performance. Amano et al. (2020) used a rehabilitation robot with arm-weight support, electrical stimulation, and vibration during reaching exercise in individuals with chronic upper limb paresis after stroke. Rodríguez-Pérez et al. (2022) applied intensive vibratory treatment with the Amadeo robotic system in patients with subacute or chronic stroke and reported improvements in sensation and upper limb function. Yeh et al. (2021) investigated robot-aided somatosensory training with vibrotactile feedback and found improvements in proprioception and motor function in stroke survivors. In these studies, vibration was not merely an isolated stimulus, but was incorporated into repeated movement practice to enrich sensory feedback during robot-assisted training.

VTS was also integrated with VR-based rehabilitation. Lin et al. (2016) developed a vibration-assisted glove that provided task-synchronized finger cues during VR games, although testing was limited to four healthy participants. Bae and Park (2023) developed an immersive gesture-controlled rhythm game with wrist vibrotactile feedback (VTF) and observed task-related functional near-infrared spectroscopy (fNIRS) activation in 11 healthy adults and one stroke survivor; however, the VTS-specific contribution was not isolated. Batista et al. (2024) further combined embodied VR, MI-BCI, and VTF and reported stronger alpha-band event-related desynchronization (ERD) with vibrotactile feedback, whereas the lateralization index did not differ significantly across conditions. Thus, current VR evidence primarily supports technical feasibility and sensorimotor engagement rather than established clinical efficacy.

Mirror-related applications mainly involved MVF combined with VTS or clinical comparison between mirror therapy and vibration therapy. Ding et al. (2020) examined MVF combined with VTS in healthy participants and found that the combined intervention promoted embodiment perception and modulated electroencephalography (EEG) sensorimotor responses. Oliveira et al. (2018) compared mirror therapy, vibration therapy and conventional physiotherapy in individuals with post-stroke hemiparesis and assessed upper limb functional outcomes. Although the number of studies in this direction was limited, these findings suggest that VTS may strengthen mirror-related rehabilitation by adding tactile input to visual feedback, thereby potentially enhancing embodiment and sensorimotor engagement (Thieme et al., 2013).

Another important application direction was the use of VTS within BCI paradigms. Shu et al. (2018) investigated tactile stimulation applied to the wrist and found that it improved sensorimotor rhythm (SMR)-based BCI performance in stroke patients by increasing classification accuracy and enhancing sensorimotor rhythm modulation. In a related study, Shu et al. (2017) examined tactile stimulation during MI and motor attempt (MA) tasks in a mixed sample of stroke survivors and healthy participants and reported that VTS improved MI-based BCI performance, with potential benefits for stroke rehabilitation. Grigorev et al. (2021) used BCI-based vibrotactile neurofeedback training and reported enhanced mu-rhythm desynchronization over contralateral motor cortex together with increased motor cortical excitability during MI. Zhang et al. (2022) investigated bilateral phase-dependent closed-loop vibration stimulation combined with an MI paradigm and reported enhanced sensorimotor cortical activation and functional connectivity. Ramu and Lakshminarayanan (2023) and Lakshminarayanan et al. (2024) explored VTS for enhancing MI and BCI-controlled rehabilitation prototypes. Yakovlev et al. (2023) further showed that real tactile stimulation and tactile imagery both induced contralateral mu-band ERD, whereas beta-band ERD was mainly observed during real stimulation. Zhang et al. (2026) evaluated a vibrotactile-assisted brain-controlled soft rehabilitation glove in 23 healthy participants and five people with stroke; VTS enhanced sensorimotor activation and improved online MI decoding mainly in participants with poorer baseline decoding performance, while the stroke subgroup showed strengthened bilateral sensorimotor activation. Chen et al. (2019) examined age-related EEG responses to VTS and found that older adults exhibited altered cortical activation patterns, reduced EEG power, and significantly lower BCI classification accuracy than younger adults. Together, these studies suggest that VTS may provide somatosensory feedback for imagined or attempted movement and may improve the stability or discriminability of BCI-related sensorimotor activity (Cervera et al., 2018; Fleury et al., 2020; Khan et al., 2020; Mansour et al., 2022; Yang et al., 2022).

Positive findings were reported across robot-assisted and BCI-related paradigms, particularly for sensorimotor performance, proprioception, cortical activation, and BCI-related outcomes. However, these bodies of evidence were dominated by small, heterogeneous, or healthy-participant studies, and the available data do not support comparative-effectiveness conclusions across application modes. The observed pattern is compatible with the hypothesis that pairing VTS with active, task-oriented training may be advantageous, but direct comparative evidence remains insufficient.

Stimulation parameters also varied substantially across the included studies. Reported frequencies included 27 Hz, 35 Hz, 60 Hz, 90 Hz, 100 Hz, 150 Hz, 200 Hz, random-frequency vibration filtered up to 500 Hz and actuator-dependent frequency ranges. Stimulation sites included the wrist, hand, fingers, fingertips, forearm, upper arm and muscle- or tendon-adjacent regions. Amplitude and intensity were inconsistently reported, with some studies adjusting stimulation according to sensory threshold, comfort or perceptibility. Treatment duration also ranged from single-session experimental stimulation to repeated clinical or home-based interventions. Therefore, although the included studies demonstrate diverse uses of VTS, the heterogeneity of stimulation parameters limits direct comparison across studies and prevents firm conclusions about the optimal frequency, intensity, site, timing or dose.

### 3.5 Clinical and functional outcomes

Clinical and functional outcomes were assessed using motor, sensory, spasticity-related and activity-based measures. Motor outcomes included the Fugl-Meyer Assessment for Upper Extremity (FMA-UE), Action Research Arm Test (ARAT), Wolf Motor Function Test (WMFT), Box and Block Test (BBT), grip strength, pinch strength, range of motion, reaching performance, grasping quality, movement smoothness and task completion measures. Sensory outcomes included Semmes-Weinstein monofilament testing (SWMT), proprioceptive thresholds and position sense. Functional outcomes included the Motor Activity Log (MAL), Stroke Impact Scale (SIS), adherence measures and observed affected upper limb activity.

Several stroke-related studies reported potential improvements in upper-limb motor performance, particularly when vibration was combined with active movement practice, robotic rehabilitation, or home-based exercise. Amano et al. (2020) observed improvements in FMA-UE, ARAT, and elbow active range after a multimodal robot-assisted program, although the independent contribution of vibration could not be isolated. Rodríguez-Pérez et al. (2022) reported improvements in sensation and motor function after robot-integrated vibration plus conventional rehabilitation. Seo et al. (2019) reported improved BBT performance after subsensory wrist stimulation during task-practice therapy, whereas Wu et al. (2017) found only modest, non-significant functional changes after glove-based training. These findings remain preliminary because of small samples, heterogeneous comparators, and frequent use of multimodal interventions.

Sensory and proprioceptive findings were mainly reported in studies using robotic, wearable, or supplementary feedback systems. Yeh et al. (2021) reported improved wrist position-sense thresholds after robot-aided somatosensory training in stroke survivors, while Cuppone et al. (2016) showed enhanced proprioceptive performance in healthy participants after robot-assisted training with vibrotactile feedback. Seim et al. (2021) also reported improved sensory outcomes after glove-based VTS in chronic stroke survivors.

Spasticity was examined in several clinical studies involving stroke survivors. Seim et al. (2021) reported reduced finger spasticity and improved sensory outcomes after glove-based VTS in chronic stroke survivors. In another study, Seim et al. (2023) also compared acute muscle and skin stimulation methods in individuals with post-stroke hand spasticity and found that cutaneous fingertip stimulation produced significant reductions in spasticity measures. Oliveira et al. (2018) and Amano et al. (2020) also assessed spasticity-related outcomes, reporting these findings alongside motor function and range-of-motion measures.

Functional use was evaluated through observational measures, adherence outcomes and self-reported functional scales. Signal et al. (2020) reported that haptic nudges increased the odds of affected upper limb movement during inpatient stroke rehabilitation. Scronce et al. (2023) examined home exercise adherence combined with TheraBracelet use, while other studies assessed MAL, SIS or functional task performance or user engagement. Overall, the included studies reported potential benefits for motor performance, sensory function, proprioception, spasticity, and functional use. However, the interpretation of these findings is limited by substantial heterogeneity in study designs, intervention protocols, and outcome measures across the included studies. The use of different outcome measures, including impairment-based assessments (e.g., FMA-UE), activity-based tests (e.g., ARAT, WMFT, and BBT), and patient-reported functional measures (e.g., MAL and SIS), may further limit the direct comparability of treatment effects across studies.

### 3.6 Neurophysiological outcomes

Neurophysiological outcomes were assessed mainly in BCI, MI, EEG, fNIRS, and sensorimotor feedback studies. Outcome measures included event-related desynchronization and synchronization (ERD/ERS), SMR modulation, BCI classification accuracy, EEG connectivity, fNIRS hemodynamic responses, motor evoked potentials and cortical activation patterns.

Several studies reported that VTS enhanced MI-related or BCI-related neural activity. Shu et al. (2018) found that tactile stimulation improved SMR-based BCI performance in stroke patients and enhanced cortical activation during MI in a mixed healthy-stroke sample. Zhang et al. (2022) reported that phase-dependent closed-loop vibration increased sensorimotor cortical activation and functional connectivity during MI. Batista et al. (2024) found that VR combined with vibrotactile feedback produced stronger alpha-band ERD during MI conditions.

Other studies examined cortical responses during vibrotactile-enhanced hand rehabilitation or sensory stimulation. Du et al. (2022) reported stronger sensorimotor activation and connectivity during vibrotactile-enhanced hand rehabilitation tasks in healthy participants. Bae and Park (2023) used fNIRS to examine an immersive VR hand rehabilitation system with vibrotactile feedback. Alouit et al. (2025) and Chen et al. (2019) investigated EEG responses to vibrotactile finger or wrist stimulation in healthy participants. Adham et al. (2024) further suggested that vibratory feedback may enhance recruitment of sensorimotor areas during MI, particularly when combined with visual feedback.

Collectively, these findings indicate that VTS and vibrotactile feedback can modulate sensorimotor neural activity. However, most neurophysiological studies involved healthy participants or small experimental samples and it remains unclear whether these neural changes translate into sustained clinical recovery.

## 4 Discussion

This systematic review synthesized clinical evidence regarding VTS for post-stroke upper-limb rehabilitation and complementary mechanistic evidence from sensorimotor training studies. Overall, clinical studies suggest that VTS may serve as a promising adjunctive approach for improving upper-limb rehabilitation outcomes after stroke, while mechanistic studies provide insights into how enhanced sensory input may influence sensorimotor processing, cortical activity, and technology-assisted rehabilitation (Grant et al., 2018; Yilmazer et al., 2019). However, the current evidence remains preliminary because of substantial heterogeneity in study populations, intervention paradigms, stimulation parameters, outcome measures, and methodological quality.

The most clinically relevant findings appeared in studies where VTS was combined with active rehabilitation approaches, including task-oriented training, robotic rehabilitation, VR, BCI systems, and wearable home-based devices. In these contexts, VTS may function not only as passive sensory stimulation but also as augmented feedback that supports movement awareness, motor intention, and repetitive task practice. Nevertheless, many included studies were pilot, feasibility, crossover, or single-session experimental studies, and several involved healthy participants rather than stroke survivors. Therefore, although the findings are encouraging, further high-quality clinical trials are needed before VTS can be recommended as a standardized rehabilitation intervention for upper limb rehabilitation after stroke.

### 4.1 Mechanisms of VTS for upper limb rehabilitation

VTS may support upper limb rehabilitation through an integrated sensorimotor mechanism that links peripheral afferent input, cortical modulation and task-related motor learning. At the peripheral level, vibration applied to the fingers, hand, wrist, or forearm may activate sensory receptors in the skin. Depending on the stimulation site and intensity, it may also stimulate sensory pathways related to muscles and joints (Johansson and Flanagan, 2009; Proske and Gandevia, 2012; Basteris et al., 2014). This additional sensory input can increase the salience of the affected limb, improve awareness of limb position and movement and provide the CNS with richer feedback for movement planning and correction (Meyer et al., 2014; Borich et al., 2015; Serrada et al., 2019; Stoykov et al., 2022). This mechanism is particularly relevant after stroke, because tactile and proprioceptive impairments may reduce sensory feedback from the paretic limb, disrupt sensorimotor integration and contribute to learned non-use.

At the cortical level, vibrotactile input may influence sensorimotor excitability and neuroplasticity by repeatedly activating sensory pathways that interact with motor-related cortical regions (Du et al., 2022; Schranz et al., 2022; de Freitas Zanona et al., 2023; Alouit et al., 2025). Studies using EEG, fNIRS, MI and BCI paradigms reported changes in ERD/ERS, SMR modulation, cortical activation, functional connectivity and BCI classification performance after VTS or vibrotactile feedback (Shu et al., 2017; 2018; Chen et al., 2019; Grigorev et al., 2021; Adham et al., 2024). These findings suggest that VTS can increase sensorimotor engagement and may help reinforce the coupling between sensory feedback and motor intention (Edwards et al., 2019; Asan et al., 2022). However, these neurophysiological findings should be interpreted cautiously, because many studies involved healthy participants or short-term experimental tasks. Therefore, cortical activation should be regarded as mechanistic support for VTS rather than direct evidence that VTS alone produces sustained clinical recovery.

A further mechanism is the use of VTS as augmented feedback during active motor learning. When vibration is delivered in relation to limb position, movement error, task performance, or MI, it may help participants associate motor intention with sensory consequences. In robot-assisted training, vibration can complement mechanical assistance by conveying movement quality, position, or task state (Cuppone et al., 2016; Amano et al., 2020; Rodríguez-Pérez et al., 2022; Yeh et al., 2021). In VR, task-synchronized VTS may strengthen embodiment and sensorimotor engagement (Lin et al., 2016; Bae and Park, 2023; Batista et al., 2024). In BCI paradigms, vibrotactile feedback may provide a sensory consequence when overt movement is limited, thereby reinforcing the relationship between imagined or attempted movement and somatosensory input (Shu et al., 2017; 2018; Grigorev et al., 2021; Zhang et al., 2022; Ramu and Lakshminarayanan, 2023; Batista et al., 2024; Lakshminarayanan et al., 2024).

These mechanisms also suggest that VTS is unlikely to be equally effective across all intervention contexts. Passive stimulation may mainly increase afferent input and sensory awareness, whereas task-contingent stimulation may provide information that supports error correction, motor learning, and engagement (Bolognini et al., 2016; Risi et al., 2019; Asan et al., 2022). Wearable haptic prompting has a different behavioral role by encouraging affected-arm use during daily activities. The therapeutic effect of VTS may therefore depend not only on the physical stimulus but also on the information carried by the signal and its temporal relationship to rehabilitation practice.

The effects of VTS are also likely to be parameter-dependent. Frequency, amplitude, stimulation site, timing, duration and treatment dose may influence whether vibration primarily provides tactile cueing, proprioceptive enhancement, attentional prompting, or task-related feedback. Across the included studies, stimulation frequencies, sites, and device designs varied substantially and amplitude or perceived intensity was often incompletely reported. This heterogeneity makes it difficult to identify an optimal stimulation protocol or to compare findings across studies. Future research should therefore report VTS parameters in sufficient detail and examine whether different parameter settings produce distinct sensory, motor, neurophysiological, usability and clinical outcomes.

### 4.2 Clinical implications and translational prospects

**FIGURE 4:**
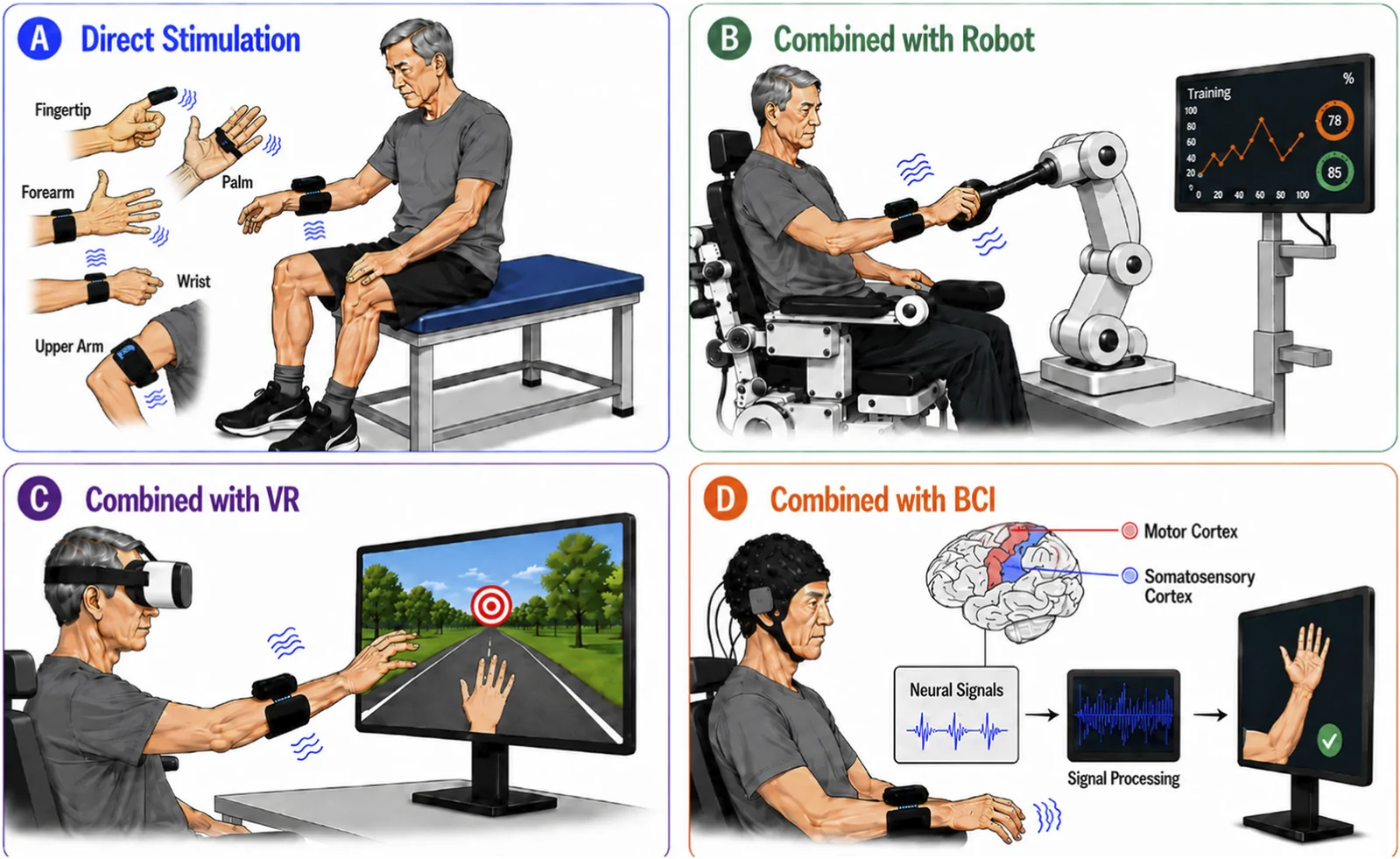
Major application modes of vibration stimulation in rehabilitation-related training: **(A)** direct stimulation; integration with **(B)** robotic systems, **(C)** virtual reality, and **(D)** brain–computer interfaces.

From a clinical perspective, VTS is an attractive adjunctive strategy for upper limb rehabilitation because it is non-invasive, relatively low-cost, wearable and easy to integrate with existing rehabilitation technologies. The findings of this review suggest that VTS may be most clinically useful when combined with active, repetitive and task-specific training rather than delivered as isolated passive stimulation. In robotic rehabilitation, VTS may complement mechanical assistance by adding sensory feedback about movement quality, limb position or task success. In VR and haptic environments, it may increase embodiment and task engagement. In BCI training, it may provide sensory feedback when overt movement is limited and may help patients associate motor intention with sensory consequences. Figure 4 summarizes four principal ways in which vibration stimulation has been incorporated into rehabilitation-related training: direct stimulation and integration with VR, robotic systems, and BCI paradigms. This framework highlights that vibration can function as background sensory input, a task-related cue, real-time performance feedback, or a component of an interactive rehabilitation loop.

The translational value of VTS may be particularly relevant for patients with sensory impairment, reduced use of the paretic limb, poor movement awareness or limited voluntary movement. Wearable vibrotactile systems and haptic nudging devices may also support home-based rehabilitation by providing repeated sensory cues outside the clinic, which may encourage use of the affected limb during daily activities. However, clinical implementation should be goal-directed. Stimulation applied to the fingers or palm may be more relevant for grasping, tactile discrimination and object manipulation, whereas wrist or forearm stimulation may be more suitable for movement cueing, limb awareness, or wearable feedback. Patient comfort, cognitive load, skin tolerance, ease of donning, device stability and adherence should be considered alongside motor outcomes.

At present, VTS should be positioned as an adjunct to conventional and technology-assisted rehabilitation rather than as a replacement for established therapy. Its value is likely to depend on whether the stimulation is paired with meaningful motor practice, whether the feedback is understandable to the patient, and whether the device can be used consistently in clinical or home settings. Future translational studies should therefore evaluate not only impairment-level outcomes but also usability, adherence, safety, therapist workload and real-world affected arm use.

### 4.3 Effectiveness outcomes and evidence gaps

The effectiveness outcomes reported in the included studies can be grouped into clinical motor outcomes, sensory and proprioceptive outcomes, spasticity-related outcomes, functional use and neurophysiological outcomes. Overall, the most encouraging findings were observed when VTS or vibrotactile feedback was combined with active rehabilitation approaches such as task-oriented practice, robotic training, VR, MI or wearable home-based prompting. Reported benefits included improvements in upper limb motor performance, grasping-related task performance, sensory or proprioceptive measures, spasticity, affected limb use and sensorimotor neural modulation. These findings support the potential value of VTS, but they should be interpreted cautiously because outcome measures and intervention protocols differed substantially across studies.

The current evidence is not yet strong enough to support definitive clinical recommendations. Many studies had small samples and used pilot, feasibility, crossover, case-series or single-session experimental designs. Several neurophysiological studies involved healthy participants rather than stroke survivors, which limits direct clinical generalizability. In addition, the independent contribution of VTS is often difficult to isolate because vibration was frequently embedded within multimodal interventions such as robotics, VR, exoskeletons or BCI systems. The absence of standardized stimulation protocols, inconsistent reporting of intensity and dose and limited long-term follow-up further reduce confidence in the durability and specificity of observed effects.

Future studies should use larger randomized controlled designs with clearly defined patient populations, comparator conditions and rehabilitation goals. Trials should report VTS parameters in detail, including stimulation site, frequency, amplitude or perceived intensity, timing, duration, total dose and adaptation procedures. Core outcomes should include standardized upper limb measures such as FMA-UE, ARAT, WMFT, BBT, MAL, sensory and proprioceptive assessments, spasticity measures, adverse events, comfort, adherence and follow-up assessments. For technology-assisted interventions, factorial or dismantling designs would be useful to distinguish the effects of vibrotactile feedback from the effects of the rehabilitation platform itself. Such studies are needed to determine which patients benefit most from VTS, which stimulation protocols are clinically meaningful, and whether short-term sensorimotor modulation translates into sustained functional recovery.

### 4.4 Limitations

Several limitations should be considered when interpreting the findings of this review. First, many included studies had small sample sizes and used pilot, feasibility, crossover, or single-session experimental designs. The MMAT appraisal additionally identified incomplete reporting of randomization procedures, absent or unclear outcome-assessor blinding, limited sample representativeness, and inadequate control of confounding in several reports. Second, study populations were heterogeneous and included both healthy participants and stroke survivors. This limits the direct generalizability of some findings to stroke rehabilitation. Third, intervention protocols varied substantially in terms of device type, stimulation site, frequency, amplitude, duration, timing, and treatment dose. Fourth, outcome measures were inconsistent across studies, and long-term follow-up was uncommon. Fifth, reporting of amplitude, intensity, adverse events, adherence, comfort, and user experience was often incomplete. Sixth, although multiple electronic databases were searched, the keyword strategy was focused on predefined vibrotactile related terms and may not have captured all potentially relevant studies using alternative terminology. Seventh, only studies published in English were included, which may have introduced language bias. Eighth, because the OSF registration occurred retrospectively, potential deviations between the initial review process and the finalized protocol cannot be completely excluded. These limitations prevented quantitative meta-analysis and reduced confidence in the overall strength of evidence.

## 5 Conclusion

This systematic review suggests that VTS and vibrotactile feedback may be promising adjunctive approaches for upper limb rehabilitation after stroke. Current evidence indicates potential benefits for motor performance, sensory and proprioceptive function, spasticity, functional use, and sensorimotor neural modulation, particularly when combined with active rehabilitation technologies and task-oriented training.

However, the current evidence remains limited by substantial heterogeneity in study populations, intervention protocols, stimulation sites, stimulation parameters, outcome measures, and study designs. Most included studies were small-scale or exploratory, limiting the strength and generalizability of the available evidence.

Future studies should prioritize well-designed randomized controlled trials (RCTs) with standardized stimulation protocols, consistent outcome measures, long-term follow-up, and the integration of VTS into closed-loop rehabilitation systems to establish the optimal application of VTS and its clinical effectiveness. Overall, VTS shows considerable potential as a clinically relevant adjunctive intervention, but stronger evidence is required before definitive clinical recommendations can be made.

## Data Availability

The original contributions presented in the study are included in the article/Supplementary Material, further inquiries can be directed to the corresponding authors.
